# The Use of Carbon Fibers Recovered by Pyrolysis from End-of-Life Wind Turbine Blades in Epoxy-Based Composite Panels

**DOI:** 10.3390/polym14142925

**Published:** 2022-07-19

**Authors:** Jakub Smoleń, Piotr Olesik, Jakub Jała, Andrzej Adamcio, Klaudia Kurtyka, Marcin Godzierz, Rafał Kozera, Mateusz Kozioł, Anna Boczkowska

**Affiliations:** 1 Faculty of Materials Engineering, Silesian University of Technology, Krasińskiego 8 Street, 40-019 Katowice, Poland; piotr.olesik@polsl.pl (P.O.); jakub.jala@polsl.pl (J.J.); mateusz.koziol@polsl.pl (M.K.); 2 ANMET Company, Koszarowa 6/18 Street, 67-300 Szprotawa, Poland; anmet@tlen.pl; 3 Centre of Polymer and Carbon Materials, Polish Academy of Sciences, M. Curie-Skłodowskiej 34 Street, 41-819 Zabrze, Poland; ktokarska@cmpw-pan.edu.pl (K.K.); mgodzierz@cmpw-pan.edu.pl (M.G.); 4 Faculty of Materials Science and Engineering, Warsaw University of Technology, Wołoska 141 Street, 02-507 Warsaw, Poland; rafal.kozera@pw.edu.pl (R.K.); anna.boczkowska@pw.edu.pl (A.B.)

**Keywords:** carbon fibers, polymer composites, waste, recycled materials, wind turbine blades

## Abstract

This work is devoted to evaluating the effectiveness of the recovery of carbon fibers from end-of-life wind turbine blades in the pyrolysis process, and the use of those fibers in the production of flat composite panels. The recovery of carbon fibers from wind turbine blades uses a pyrolysis process at 500–600 °C in a non-oxidizing atmosphere, in such a way that makes it possible to preserve the shape and dimensions of the fibers. Using recycled carbon fibers, flat CFRP sheets with epoxy resin matrix were produced by pressing. Seven different series of samples were tested, which differed in fiber length, fiber orientation, and pressure holding time. The results obtained on the recycled fibers were compared to the original carbon fibers, cut to corresponding lengths. Additionally, one of the series was reinforced with a biaxial fabric. The most favorable pressing parameters are empirically found to be pre-pressing 2 MPa (10 min), and further pressing at a pressure of 7 MPa until the resin completely cross-linked (about 120 min). A number of tests were carried out to demonstrate the usefulness of pyrolytic fibers, including tensile strength of carbon fibers, bending strength, SEM observations, FT-IR, and Raman spectroscopy. The tests carried out on the carbon fibers show that the pyrolysis process used leaves about 2% of the matrix on the surface of the fiber, and the tensile strength of the fibers drops by about 20% compared to the new carbon fibers. The research results show that the use of the recycled carbon fibers in the production of flat composite plates is reliable, and their mechanical properties do not differ significantly from plates made of corresponding original carbon fibers. Composite panels with the pyrolytic fibers (274 MPa) show up to a 35% higher flexural strength than similarly produced panels with the original new carbon fibers (203 MPa), which means that the panels can be used in the production of elements for footbridges, bridges, pipelines, or structural elements of buildings and roofing.

## 1. Introduction

End-of-life (EOL) wind turbine blades are a problematic waste, due to the multi-material composition applied in producing them. The main material used is a carbon-fiber-reinforced polymer (CFRP) composite (multi-materials are also used). Due to the potential for high mechanical properties of expensive carbon fibers, there has been an increasing interest in the recycling of CFRP waste in recent years. A market for CFRP composites is growing steadily. The consequence is a generation of very large amounts of problematic waste. The current state of knowledge does not define any really effective method of recycling CFRP waste. According to some sources, as much as 30–40% of carbon fibers and CFRP composites end up as waste in the manufacturing process. A good practice is to extend the service time of composite products, which can be achieved by the use of protective coatings or the use of additives (e.g., light stabilizers, flame retardants, antioxidants, etc.). Recycling of CFRP becomes problematic due to the variety of matrix materials and reinforcement form [1,2,3,4,5]. The range of carbon fibers includes short fibers, long fibers, rovings, mats, and fabrics. Carbon fibers also differ in properties such as tensile strength, modulus of elasticity, or cross-sectional diameter. These diverse factors make it impossible to identify one effective method of CFRP waste management. Very often, CFRP recycling aims to only recover carbon fibers at the expense of losing the matrix material. One of the most commonly used recycling methods is pyrolysis. Due to the high-temperature nature of the process (range 400–1000 °C), it is classified as thermal recycling. It is an effective method that allows the preservation of the properties of carbon fibers, such as up to 85–94% of their original tensile strength [6,7]. The decomposition products of the resin form byproducts such as gas, oil, and char [8]. The pyrolysis method is competitive with other methods of recycling, such as the fluidized bed method, mechanical recycling, and chemical recycling [9,10,11,12].

This article presents an attempt to manage CFRP waste from dismantled wind turbine blades in Central and Western Europe. Pyrolysis was carried out as a form of thermal recycling of CFRP composites in order to recover carbon fibers with a length of up to 3000 mm. The chopped carbon fibers were mixed with epoxy resin and hot pressed under various conditions, in the form of a plate with dimensions of 300 × 200 mm. A series of seven panels was made, including 15 mm, 75 mm, and 300 mm (continuous) long fiber samples. For comparison, samples were made from both the pyrolytic fibers and from corresponding new fibers. The paper presents the evaluation of the mechanical properties in a static three-point bending test. The research results show the high application potential of composite panels containing recycled carbon fiber, e.g., in the construction, automotive, and architectural sectors.

## 2. Materials and Methods

### 2.1. Materials

The materials used for the research process were elements of a wind turbine blade. Several dozen meters long blades were cut into elements with a maximum length of 3 m, both for easier transport and because the pyrolysis process was limited by the dimensions of the furnace chamber. They were also devoid of additional elements, such as metal or foam inserts. Large pieces of CFRP composites were pyrolyzed in an electric furnace. The temperature of the process ranged from 500 to 600 °C, and the pyrolysis process took place in anaerobic conditions. Pyrolysis resulted in a 1–2% matrix residue on the surface of the fibers. After the pyrolysis process was completed, the carbon fibers were cut to lengths of 15 mm, 75 mm, and over 300 mm using a cutting guillotine. The matrix material of the composite was epoxy resin Epidian 652 (Sarzyna Chemical, Nowa Sarzyna, Poland) cured with MTB (Sarzyna Chemical, Nowa Sarzyna, Poland) in a weight ratio of 100:37. Seven series of samples were produced, as described in Table 1. Pressing parameters were selected empirically, based on previous experimental tests. Series 1 and 2 were conducted in different pressures to evaluate the effectiveness of the pressure retention to completely cross-link the resin. Experiments show the effectiveness of maintaining the pressure of 7 MPa from 10 min to hardening (about 120 min). In series 3 and 6, the original carbon fibers Tenax^®^-E HTS40 F13 24K 1600tex (Tenax, Tokio, Japan) were used to compare the results obtained in the fiber-based samples after the pyrolysis process. Additionally, in series 5, one layer of biaxial fabric SAERTEX^®^ U-CE-464 g/m^2^−1270 mm (SAERTEX, Saerbeck, Germany) was used, in order to intentionally affect the mechanical properties.

### 2.2. Preparation of Samples

**Figure 1:**
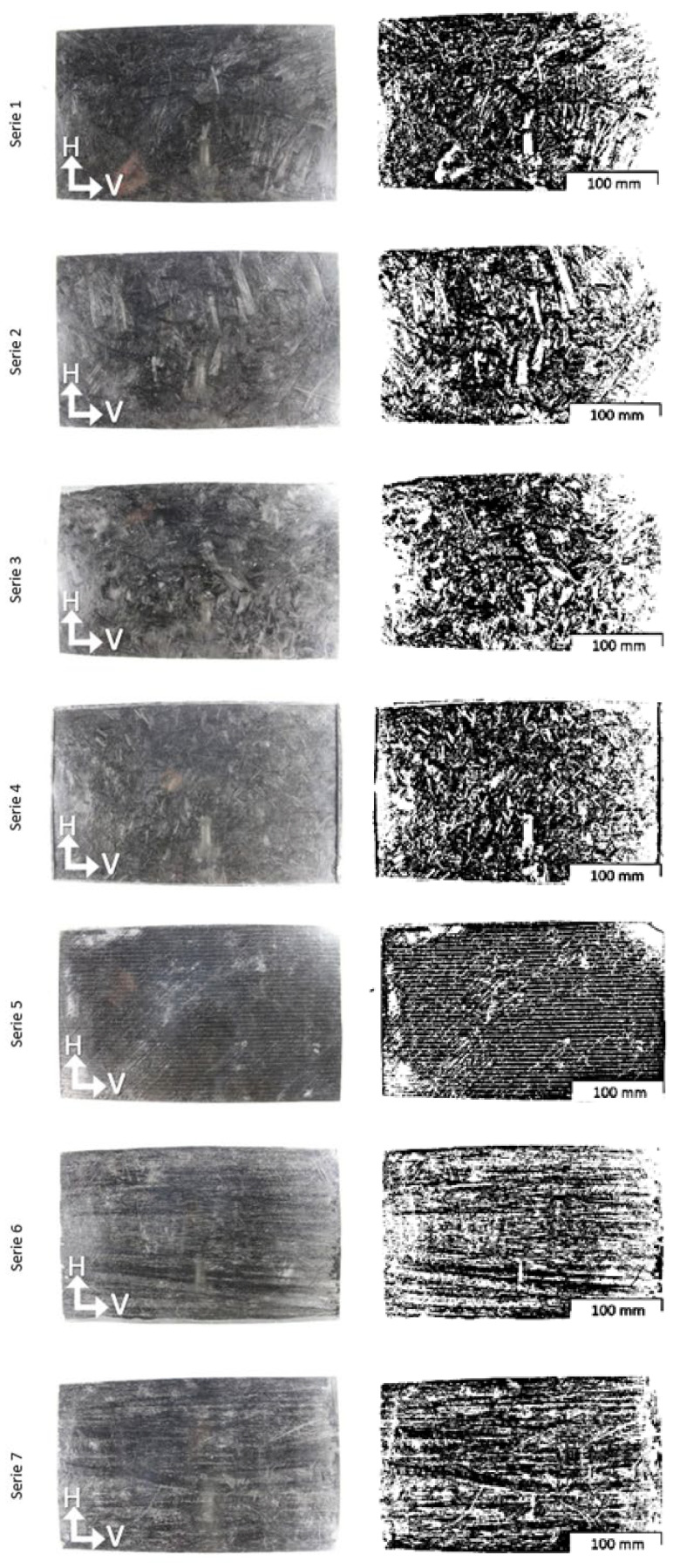
Macro-scale photographs of surfaces of sample panels: left column—raw data, right column—data after binarization, where H is bending direction along the fiber placement direction and V is bending direction perpendicular to the fiber placement direction.

The carbon fibers in series 1–5 were mixed (around 5 min) with epoxy resin in a container and then placed inside the mold. Pre-compression was carried out at 2 MPa for 10 min, then pressurized to 7 MPa, which was held for a further 10 min (series 1), or held until the resin cross-linked (series 2–7), in an ambient temperature. In series 5, a layer of biaxial fabric was additionally placed inside the mold, and then a layer of the fibers–resin mixture was applied. Samples 6–7 required a different preparation procedure. First, the fiber was placed inside the mold, then epoxy resin was poured in, and finally the samples were pressed. After the resin cured, the composite was removed from the mold, and the excess material was cut off at the edges. Then, samples with dimensions of 100 × 25 mm were cut from the plates. A total of 8 samples with bending direction perpendicular to the fiber placement direction (V) and 14 samples with bending direction along the fiber placement direction (H) were cut from each panel. The photos of the panels showing the distribution of fibers in the material are shown in Figure 1. 

### 2.3. Testing Procedure

The original carbon fiber and the pyrolysis fiber were inspected using a scanning electron microscope (Hitachi S-4200), and the SE (secondary electron) technique at 5000× magnification. Images of the samples were taken using digital photography techniques to show the distribution of fibers in the composite material, and the images were binarized to increase the clarity. The three-point static bending test of the samples was carried out in accordance with the PN-EN ISO 14125 standard. The flexural strength (R_g_), Young’s modulus (E_flex_), and bending strain corresponding with R_g_ point (ε_flex_) were tested at room temperature on the Shimadzu AGX-V machine (Japan). The support spacing was 80 mm, and the loading bar speed was 10 mm/min. Statistical analysis of the results was performed using Origin software. Obtained data were evaluated with the Grubbs test for significant outliers. After rejecting insufficient results, the means were statistically compared with ANOVA, followed by a post-hoc Tukey test.

The samples from series 2 and 3 were studied using Fourier-transform infrared (FTIR) spectroscopy, using a Thermo Scientific Nicolet 6700 spectrometer with suppressed total reflection (ATR). A Smart Orbit with a diamond crystal was used for this purpose. Intensities of the spectra were normalized relatively to the maximum recorded peak. Raman spectra were measured using a WITec Alpha M300+ Confocal Raman microscope (532 nm, 1 mW). Intensities of the spectra were normalized to the G peak (for carbon fibers), or to the maximum peak (for epoxy resins). 

In order to analyze mechanical characteristics of the material on samples from series 2 and 3, a static tensile test was performed in accordance with PN-EN ISO 527 on the INSTRON 4469 testing machine, at loading clamp speed of 5 mm/min. 

Additional tensile tests were also performed on the original carbon fibers in bundles and pyrolytic carbon fibers in accordance with ASTM D4018-99. The carbon fiber tensile test was performed on the Zwick-Roell Z006 testing machine. 

## 3. Results and Discussion

### 3.1. Surface of Fibers

**Figure 2:**
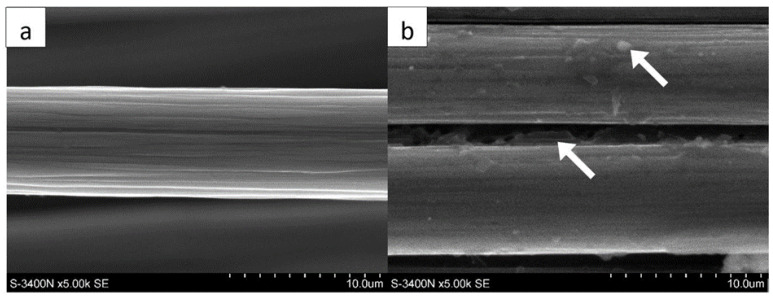
SEM images of studied carbon fibers: (**a**) the original carbon fiber, (**b**) the pyrolytic carbon fibers (Hitachi S-4200, SE technique, magnification 5000×).

Figure 2 shows the original (Figure 2a) and the pyrolytic (Figure 2b) fibers. The surface of the new fibers is devoid of defects, e.g., holes and gaps, while the recycled fibers have carbonized epoxy matrix residues, which are marked with arrows in Figure 2b. Despite visible residual resin on the fiber, the fiber itself appears undamaged. The irregularities in the fiber surface cause the surface expansion, and an increase in the matrix–fiber interface. The fiber diameter before and after the pyrolysis process does not change, and is about 7 µm. 

### 3.2. Three-Point Bending Tests

**Figure 3:**
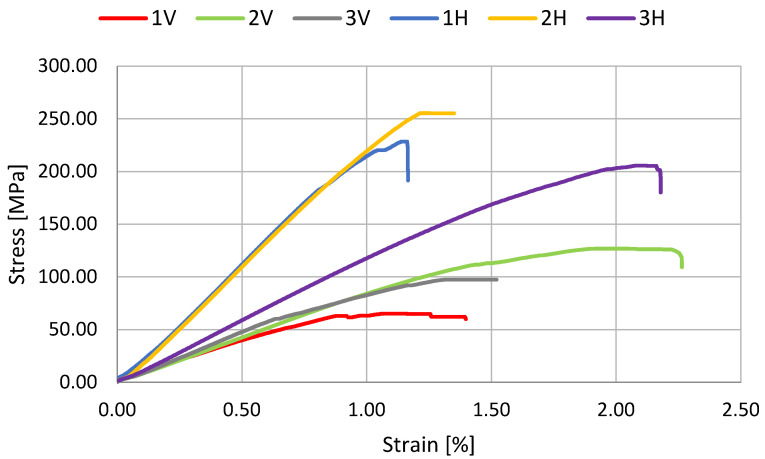
Representative curves for samples with 75 mm long carbon fibers.

**Figure 4:**
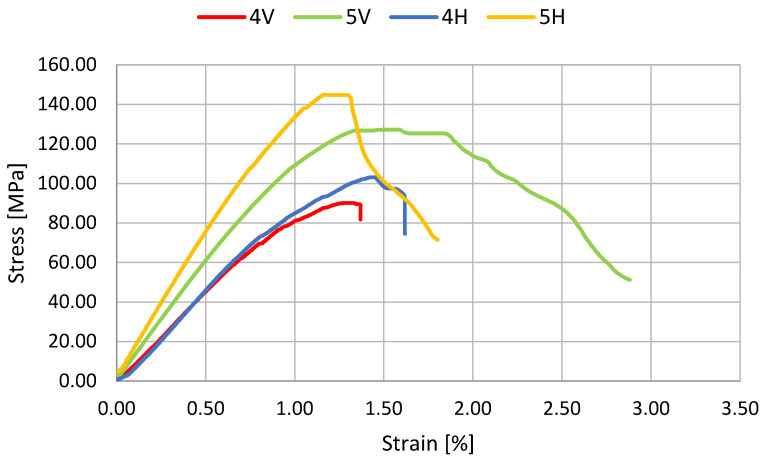
Representative curves for samples with 15 mm long carbon fibers.

**Figure 5:**
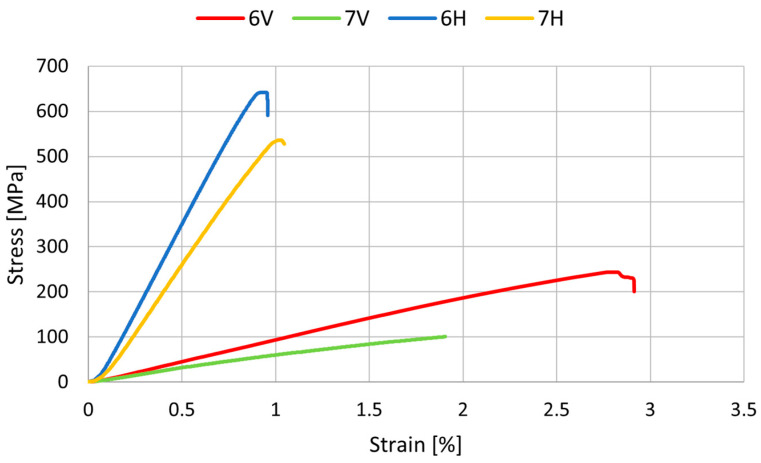
Representative curves for samples with 300 mm long carbon fibers.

The results of the three-point bending test are shown in Table 2 and in Figure 3, Figure 4 and Figure 5. The statistical analysis, which includes rejecting outliers and mean value comparison, reveals some connections between samples. First, and most important, is that the composite reinforced with carbon fiber after the pyrolysis process shows significant improvement in flexural strength. For samples 2H and 6H, the increase in R_g_ reaches 35.3% (~70 MPa) and 14.9% (~90 Mpa), respectively, in comparison with the corresponding samples reinforced with the original carbon fibers (3H and 7H). Such phenomena may be connected with better surface interaction with the curing resin. The hypothesis suggests that during the composite curing process, the resin residues on the fiber surface, representing good adhesion to the fibers (maybe due to more cross-linked structure), result in a more developed interface. 

The impact of manufacturing conditions on the materials’ properties was also tested. The time of applied pressure does not significantly impact the material strength (samples 1H and 2H). However, as expected, the fiber’s length has a significant impact on material anisotropy. For series 1, 2, 3, 6, and 7 there is significant difference between samples that were cut off into alternative perpendicular directions. During the pouring of the resin/fibers mixture into the mold, the longer fibers (see Table 1) partially, or even fully, orient themselves, which has an effect on the flexural strength and Young’s modulus of the composites. The direction of the fibers was observed visually. However, the bending strain only significantly differs when fibers are at least 300 mm long. Finally, the samples from series 4 and 5 exhibit isotropic properties, and the usage of outer layers of biaxial fabric leads to significantly higher mechanical properties. This case suggests that reinforcing a mass based on recycled carbon fiber with additional carbon fabrics is advantageous.

### 3.3. FT-IR and Raman Investigation

**Figure 6:**
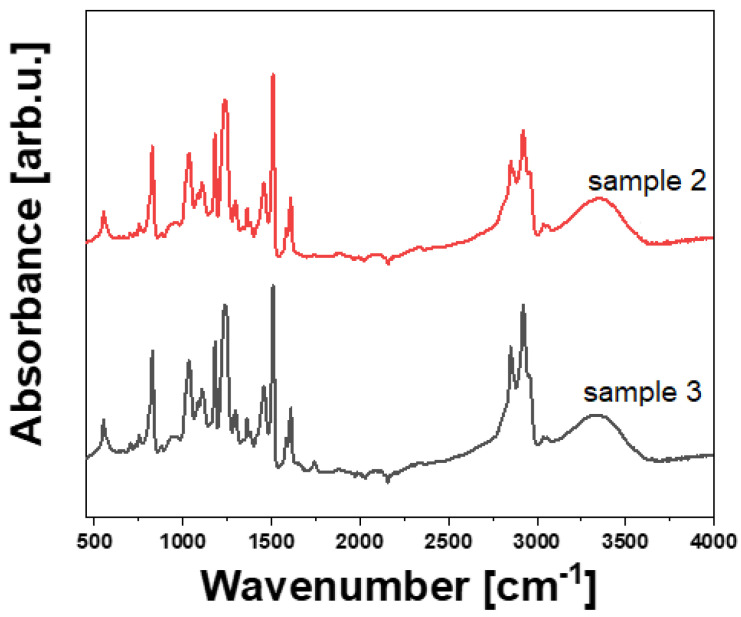
FT--IR spectra of the examined CFRP laminates.

**Figure 7:**
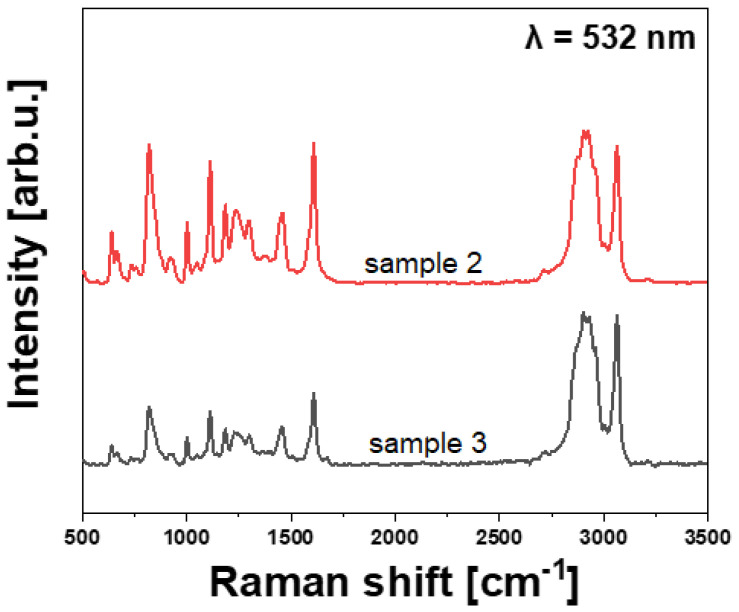
Raman spectra of the examined CFRP laminates in the epoxy matrix regions.

**Figure 8:**
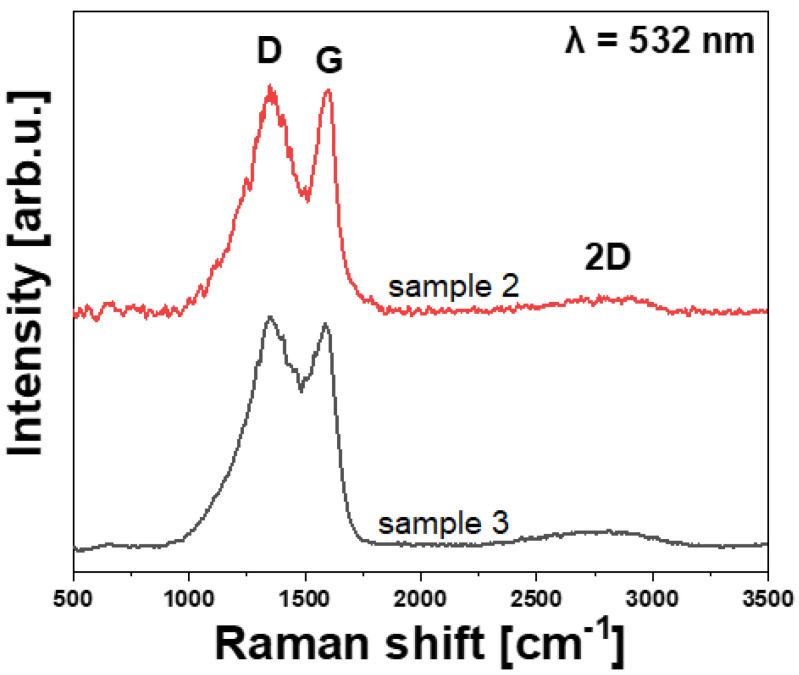
Raman spectra of the examined CFRP laminates in the carbon fibers regions.

The results of FT-IR and Raman spectroscopy studies aimed to explain the variations observed in the flexural properties of the laminates. The results are presented in Figure 6, Figure 7 and Figure 8, and Table 3 and Table 4. It is seen that the FT-IR and Raman spectra of the epoxy resins region from the examined CFRP laminates are similar, which suggests a similar chemical structure [13,14]. Table 3 and Table 4 list characteristic absorption bands detected in both samples. It should be noted that most of the detected peaks correspond to the chemical structure of epoxy resin used in this study, but also a very fine peak corresponding to the presence of the carbonyl group is detected in both FT-IR spectra. It might be a result of the high curing temperature, as suggested by Krauklis et al. [15] and Ramirez-Herrera et al. [14]. The authors also stated that a higher curing temperature results in higher strength of CFRP laminates reinforced with bidirectional woven carbon fabric. Moreover, the surface of the pyrolyzed fibers is, in general, much more oxidized than the surface of the original new fibers—see the outer chemical groups set in Table 3. It has a doubtlessly advantageous effect on the creation of strong bonds between the fibers and the matrix polymer.

Figure 8 presents Raman spectra typically acquired for carbon fibers used in laminates 2 and 3. Raman spectra show two characteristic bands: D (1349 cm^−1^) and G (1585/1598 cm^−1^). As can be seen, the D band is not detected, confirming that the used carbon fibers are not high modulus (highly graphitized) ones. Based on the quotient of the area under the D and G curves (Lorentz fit), it is possible to determine the degree of disturbance of the symmetrical structure of the sp2 of the tested materials [16,17]. The greater the value of D/G, the more defective the symmetry of the structure, resulting in a decrease in the fibers tensile strength and tensile modulus, as proposed by Qian et al. [16]. The intensity of the D/G ratio is 1.82 and 1.94 for samples 2 and 3, respectively, and is in good agreement with mechanical tests of carbon fibers (see Table 5).

### 3.4. Failure Behavior of Tested Laminates

**Figure 9:**
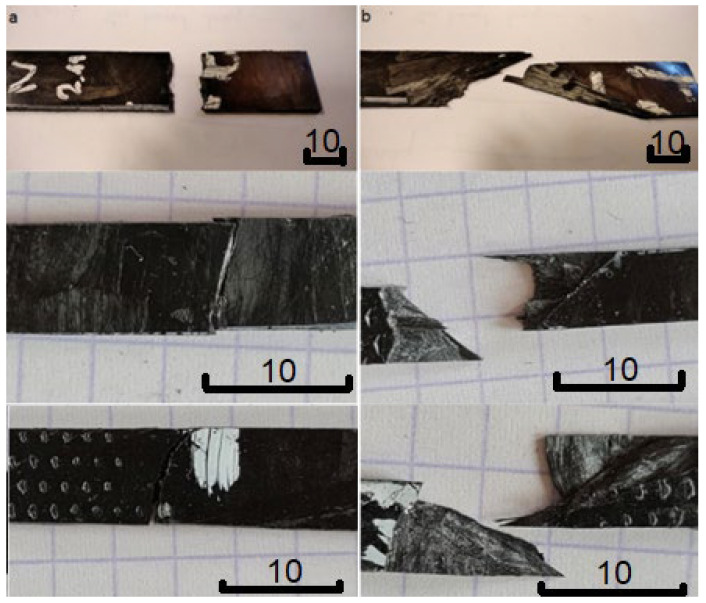
Destruction of composite samples after tensile tests: (**a**) representative samples of the laminate reinforced with the pyrolytic fibers (series 2 from Table 1), (**b**) representative samples of the laminate reinforced with the original carbon fibers (series 3 from Table 1).

The tensile tests were performed in order to confirm the trends observed for the flexural properties of studied laminates and, mainly, for performing analysis of failure behavior. It is very important for evaluation of the pyrolyzed fibers performance within the material. The tensile strength of the original carbon fibers is 2268 +/− 133 MPa, while the tensile strength of the pyrolytic carbon fibers is 1842 +/− 312 MPa. The decrease in the strength of carbon fibers by less than 20% is a satisfactory result, especially for the flexural strength of the tested composite panels with chopped fibers that show a different tendency. The increase in flexural strength recorded in sample 2 with the pyrolytic fibers compared to sample 3 with the original carbon fibers is likely due to the different nature of the failure process. The composites with the original fibers crack as a result of evident delamination, while the composites with the pyrolytic fibers do not show significant delamination-arisen failure initiation, and the failure starts from a fine fiber bundle crack and runs through the fibers. The differences between representative samples destroyed in tensile tests are clearly visible in Figure 9.

**Figure 10:**
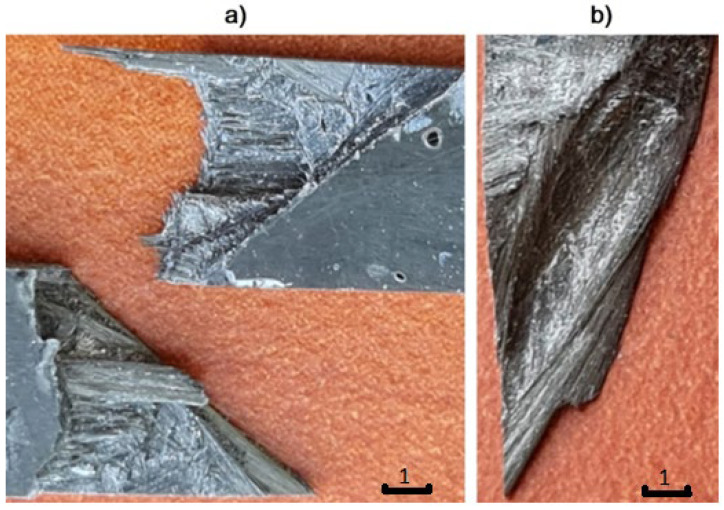
Fracture of exemplary composite samples after tensile tests—the composite reinforced with original carbon fibers: (**a**) two sides of the fractured sample, (**b**) magnified view of the fracture.

**Figure 11:**
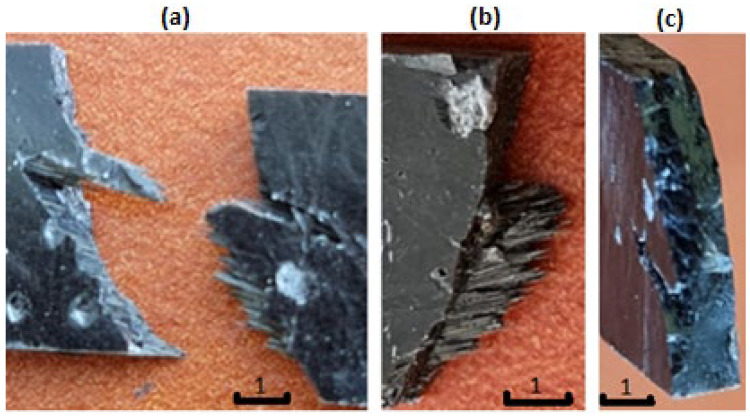
Fracture of exemplary composite samples after tensile tests—the composite reinforced with the pyrolytic carbon fibers: (**a**) two sides of the fractured sample, (**b**) view of a brittle fracture and the pulled-out fiber strands, (**c**) view of a brittle fracture with visible “holes” left by the pulled-out fiber strands.

The behavior shown in Figure 9 is repeatable for ALL samples. It is not dependent on fiber length—all types of specimens (reinforced with 15 mm, 75 mm, and 300 mm long fibers) show more elongated fracture in the case of the original reinforcement, and more typical in case of the pyrolytic one. Such behavior confirms more intensive delamination in the composites reinforced with original carbon, which probably results from relatively weak interfacial strength. The relation between the interfacial strength and the delamination susceptibility of the laminates was well proven in the past [18]. Figure 10 and Figure 11 show more detailed views of the fractures.

We can see that the fractures of the laminates reinforced with original fibers show evident decohesion of the matrix in areas between fiber strands. There are no cracked fibers visible; any fibers are pulled out from the matrix. The progress of the failure develops mainly by shear in inter-laminar and inter-strand areas. 

In the case of the laminates reinforced with pyrolytic fibers (Figure 11), we can see quite different failure effects. There is the expected brittle type of failure visible in the fractures—it proves a good load-carry efficiency of the fibers. Only part of the fracture area contains fiber strands pulled out from the opposite side. However, both mechanisms are more advantageous than those in the laminates reinforced with original carbon fibers. An irrefutable reason for the difference is better connection at interface in the case of the pyrolytic laminates. It probably results from the oxidized surface that occurs after the pyrolysis. It is a “side-effect” of the areal treatment of the fiber surface. It is a commonly used treatment in carbon-fiber-reinforced composites, with good results—the fibers become more susceptible to bonding with resin and other liquid matrices after it [19]. The second effect that improves interface connection is the enlargement of the effective surface area with remnants of the original matrix (see Figure 3). 

The obtained results show that relatively simple pyrolysis processing can be applied as an efficient method for recycling CFRP laminates. The obtained results—for the fibers as well as for the laminates—are comparable with those obtained in previous research [13,20]. On the other hand, the form of the recycled fiber strands does not enable utilizing them in a manner comparable with the neat original ones. It is partially compatible with claims in the literature [21]. In fact, taking the results of this study into account, the recycled carbon fibers can only be used for secondary, low-responsibility products. However, in such applications they may be attractive and high-quality components.

## 4. Conclusions

In this article, the possible usage of recycled carbon fibers from wind turbines was examined. The presented research leads to following conclusions:

- The pyrolysis process decreases the strength of carbon fiber by about 20%;
- After the pyrolysis process, there are resin residues on the fiber surface;
- The Raman and FT-IR spectra show that after pyrolysis, the carbon fiber structure is slightly degraded, but the surface is more oxidized;
- The laminates with recycled carbon show a 35% higher flexural strength than the laminates with the original carbon fibers;
- The increase in strength is a result of a larger contact area between the fibers and the matrix.

These results shows that using recycled carbon fibers could be advantageous in polymer composites. Composite panels with pyrolytic fibers (with a flexural strength at 274 MPa) can be advantageously used in the production of elements for footbridges, bridges, pipelines, or structural elements of buildings and roofing. However, more research, with different applications and processing conditions, should be performed to recognize a whole spectrum of applicability for these valuable materials.

## Figures and Tables

**Table 1:** Description of CFRP composite panel (300 × 200 mm) series used in the study.

Sample Series	1	2	3	4	5	6	7
**Length of carbon fibers, mm**	75	75	75 *	15	15 + biaxial fabric **	>300	>300 *
**Conditions of pressing**	10 min: 2 MPa 10 min: 7 MPa	10 min: 2 MPa 120 min: 7 MPa					

* original carbon fiber: Tenax^®^-E HTS40 F13 24K 1600tex. ** original biaxial carbon fiber fabric (one layer): SAERTEX^®^ U-CE-464 g/m^2^−1270 mm.

**Table 2:** Results of three-point bending tests—designations of the series 1–7—see Table 1; H—bending direction along the fiber placement direction, V—bending direction perpendicular to the fiber placement direction.

Sample	Flexural Strength, R_g_ [MPa]	Young’s Modulus, E_flex_ [GPa]	Bending Strain Corresponding with Rg Point (ε_flex_), [%]
**1H**	227 ± 100	19.4 ± 5.8	1.11 ± 0.33
**1V**	71.5 ± 12.6	7.73 ± 1.51	1.38 ± 0.43
**2H**	274 ± 75	21.7 ± 11.3	1.58 ± 0.27
**2V**	156 ± 80	10.11 ± 5.77	1.53 ± 0.46
**3H**	203 ± 56	14.9 ± 6	1.94 ± 0.54
**3V**	148 ± 99	9.54 ± 2.84	1.91 ± 0.78
**4H**	110 ± 21	9.55 ± 2.23	1.23 ± 0.2
**4V**	89 ± 16	10.1 ± 3.3	1.21 ± 0.33
**5H**	154 ± 40	14.9 ± 3.1	1.35 ± 0.29
**5V**	135 ± 24	13.8 ± 2.3	1.35 ± 0.21
**6H**	644 ± 96	37.8 ± 5.9	0.91 ± 0.07
**6V**	239 ± 69	8.37 ± 1.91	2.99 ± 0.42
**7H**	560 ± 76	29.6 ± 8	0.92 ± 0.07
**7V**	143 ± 10	6.89 ± 0.58	3.42 ± 0.61

**Table 3:** IR absorption bands of the examined CFRP laminates.

Wavenumber (cm^−1^)	Band Assignment
~550	−−C−H/−N−H, bending
~750	=C−H/C−H, aromatic ring
820	−C−O−C, oxirane, stretching
1030	−C−O−C, ethers, stretching
1080–1100	−O−C−C, stretching
1180–1240	−C−C−O−C, stretching
1360–1460	−CH_2_−, −CH_3_−, bending
1508–1608	−C=C−H, aromatic, stretching
1740	−C=O, carbonyl group
2850	−CH_2_−, −CH_3_−, symmetric, stretching
2920–2960	−CH_2_−, −CH_3_−, asymmetric, stretching
3030–3050	=C−H, aromatic, stretching
3340	−OH, stretching

**Table 4:** Raman bands assignments for the examined CFRP laminates.

Band Position (cm^−1^)	Assignments
641–668	Aromatic ring vibrations (p−substituted benzene); aromatic C−H out of plane deformation
735–762	C−C skeletal
819	Out of plane bending of aromatic C−H
916–933	Epoxy group
1002	Epoxy group
1114	Aromatic C−H stretching and in plane deformation
1186	C−O stretching vibration
1237–1300	C−O stretching vibration ether bridge
1456	Stretching vibration of benzene rings
1610	C−C stretching vibration of aromatic; C−O stretching vibration of amide; skeletal vibrations of C=C double bonds in aromatic ring

**Table 5:** Tensile strength of carbon fibers [MPa].

**Original carbon fiber**	2274	2317	2069	2088	2391	2353	2357	2147	2419	**2268**
**Pyrolyzed carbon fiber**	2039	2044	2164	1898	1711	1829	1090	1881	1924	**1842**

## Data Availability

Not applicable.

## List of Nomenclature

R_g_	Flexural strength: MPa
E_flex_	Young’s modulus
ε_flex_	Bending strain corresponding with R_g_ point
λ	Wavelength
